# Sentinel surveillance of influenza-like illness in the Central African Republic, 2010–2015

**DOI:** 10.1186/s13690-017-0229-1

**Published:** 2017-10-05

**Authors:** Alexandre Manirakiza, Marie-Yvette Batoumbou Ketta, Ulrich Vickos, Giscard Francis Komoyo, Sandra Garba-ouangole, Colette Bangue, Edgar Djimbele, Ombretta Pasotti, Eugene Kanga, Eloi Nicaise Mboufoungou, Brice Martial Yambiyo, Kathleen Victoir, Jean-Chrysostome Gody, Mirdad Kazanji, Emmanuel Nakoune

**Affiliations:** 1 Institut Pasteur of Bangui, Epidemiology service, PO Box 923, Bangui, Central African Republic; 2 Institut Pasteur of Bangui, Virology department, PO Box 923, Bangui, Central African Republic; 3 Complexe Pédiatrique de Bangui, Ministry of Health, PO Box 883, Bangui, Central African Republic; 4 Emergency International, Representation in Bangui, Bangui, Central African Republic; 5 Hopital de district de Bossembele, Ministry of Health, Bangui, PO Box 883, Central African Republic; 6 Hôpital secondaire de Boali, Ministry of Health, Bangui, PO Box 883, Central African Republic; 7 International Division, Institut Pasteur, Paris, France; 8 Institut Pasteur de Cayenne, 23 Avenue Pasteur, BP 6010, 97306 Cayenne Cedex, Guyane France

**Keywords:** Influenza virus, Sentinel surveillance, Central African Republic

## Abstract

**Background:**

Influenza-like illness (ILI) is an important public health problem worldwide. In the Central African Republic, acute infectious diseases are the commonest reason for consultation. The Institut Pasteur of Bangui set up a surveillance network in 2008 to monitor the circulation of influenza viruses. We report the results of use of this surveillance system during the period 2010–2015.

**Methods:**

The first surveillance centre covered Bangui, the capital of the country, and neighbouring areas and epidemiological data on syndromes similar to ILI. Throat and nasopharyngeal swab samples are transmitted weekly to the Institut Pasteur of Bangui, where real-time and multiplex reverse transcription polymerase chain reaction are used to detect and subtype influenza A (H1N1 and H3N2) and B viruses. The demographic characteristics of all patients and of positive cases according to age and the seasonal patterns of influenza virus circulation were analysed.

**Results:**

Between January 2010 and December 2015, 5385 throat swabs were collected; 454 (8.4%) of the samples were positive. Of these, 450 yielded at least one influenza virus and four showed co-infections. Children under the age of 5 years were the most frequently infected (257/450, 57.1%), with irregular peaks of ILI. Influenza B predominated (56.2%; *n* = 201), with 39.0% H3N2 and 16.7%H1N1pdm09. Influenza viruses were detected mainly in the rainy season (July–December).

**Conclusion:**

The sentinel surveillance site is yielding important information about the seasonality and age pattern of circulating influenza virus. Nationwide distribution of sentinel sites is warranted.

## Background

Influenza-like-illness (ILI) is a major public health problem worldwide [1]. Annual epidemics of influenza infection are estimated to result in 3–5 million cases of severe illness and 250,000–500,000 deaths. In developed countries, annual ILI epidemics infect about 10–20% of the population each season and cause febrile illnesses that range in severity from mild to debilitating and can lead in some instances to hospitalisation and even death [2]. Influenza viruses cause illness in individuals of all ages [3, 4]. Until recently, the epidemiology of ILI in developed countries was poorly understood; however, the few investigations of seasonal outbreaks in Africa reported alarmingly high case fatality rates [5–7]. A review of the literature between 1980 and 2009 showed limited data on influenza for most countries in sub-Saharan Africa [8]. In 2006, the African Network for Influenza Surveillance and Epidemiology (ANISE) was set up to generate data on the burden and epidemiology of influenza in Africa [7, 9]. The results show a substantial burden of influenza infection in Africa [10–17].

In the Central African Republic, acute respiratory infection is the commonest reason for paediatric consultation. In 2008, the Ministry of Health established a national influenza surveillance system to monitor the circulation of seasonal influenza and also to detect the emergence and spread of novel influenza strains with pandemic potential. The Institut Pasteur of Bangui was mandated to conduct surveillance and to identify influenza viruses in the laboratory. The surveillance system is financially supported by the United States Department of Health and Human Services. This paper reports the results of ILI surveillance in the Central African Republic between January 2010 and December 2015.

## Methods

### Setting

**Fig. 1:**
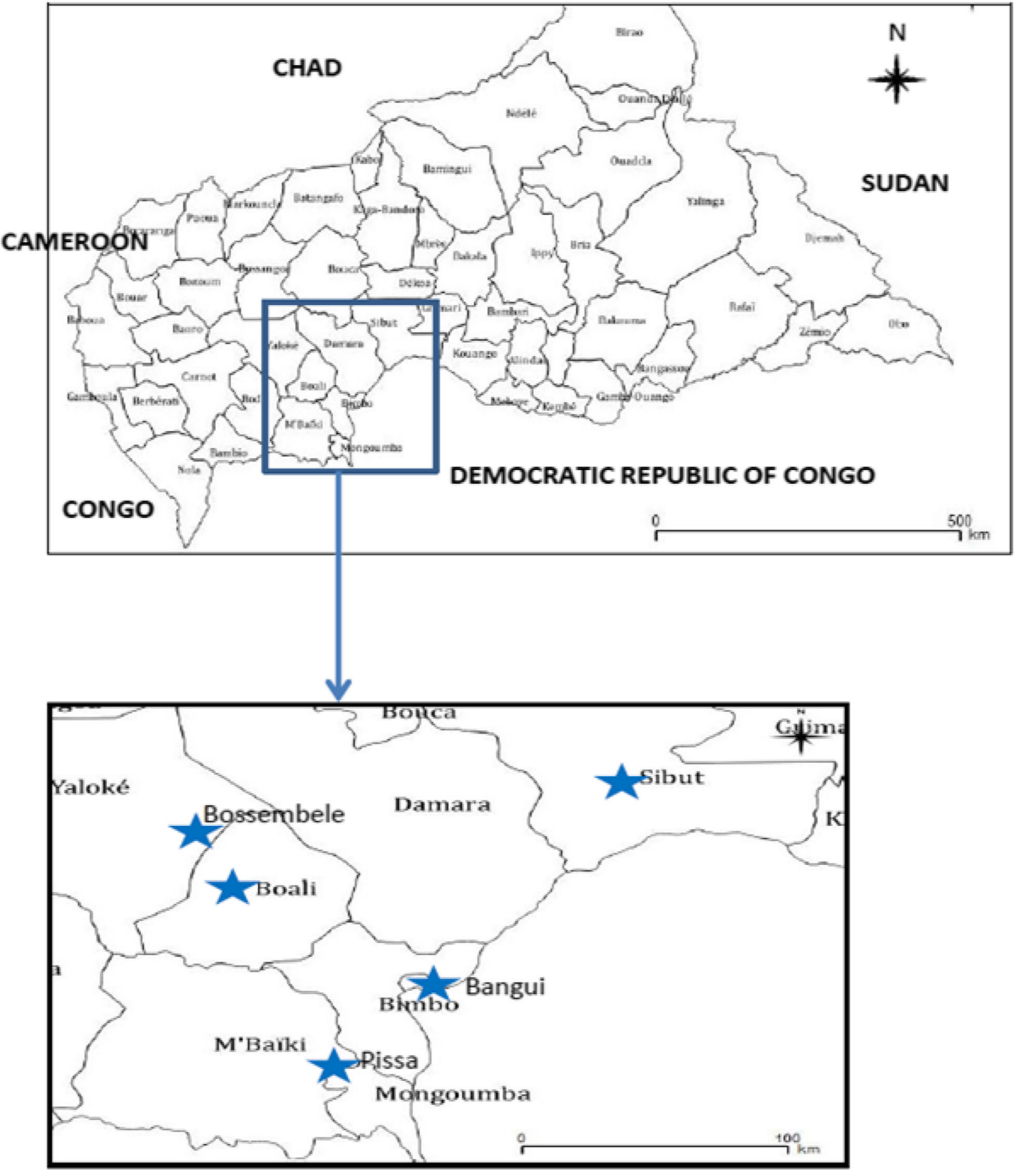
Locations of sentinel sites for surveillance of influenza-like illness, Central African Republic, 2010–2015

The ILI surveillance system is a collaborative partnership between the Ministry of Health and the National Influenza Reference Laboratory, which is hosted by the Institut Pasteur of Bangui. Current sentinel sites are located in the southern region of the country (Fig. 1), where the climate is tropical, with a long rainy season from April to November and a temperature of 19–32 °C. The first sentinel sites were established in January 2008 in Bangui, the capital of the country, at a paediatric complex that is a tertiary referral hospital; the Saint Joseph health centre, which is a private centre run by Catholic missionaries, who provide health care to very low-income populations; and a paediatric emergency centre run by an independent Italian organisation.

In 2010, surveillance was extended to public institutions of the Ministry of Health in the neighbourhood of Bangui (Pissa health centre, Boali health centre, Bossembele hospital and Sibut hospital), which were selected on the basis of their accessibility, for prompt transport of case report forms and samples to the National Influenza Reference Laboratory.

### Case definition and study population

During 2010–2013, we used the World Health Organization definition of ILI: an acute respiratory infection with fever ≥38 °C AND cough with onset within the past 10 days [18]. After 2014, the definition was changed to “an acute respiratory infection with fever ≥ 38 °C AND cough or sore with onset within the past 10 days” [19]. In both these case definitions, fever and cough may be accompanied or not by throat and general symptoms such as myalgia, prostration, headache or malaise.

The study population consisted of every outpatient presenting at any of the sentinel sites who met the ILI case definition, regardless of age or sex, who gave oral consent to participate in surveillance.

### Data and sample collection

The National Influenza Reference Laboratory provides logistical and material support to the sentinel sites, including a standardised questionnaire, swabs, viral transport media, cool boxes and ice packs. A quota of 30 samples per month was targeted from each site. The clinical personnel involved in surveillance collected information from each patient on the standardised questionnaire and recorded demographic characteristics and medical history: date of enrolment and symptom onset, gender, age and clinical symptoms. Nasopharyngeal and oropharyngeal swabs were collected, placed in a tube with a viral transport medium and stored at 2–8 °C in the sentinel site laboratory before delivery to the National Influenza Reference Laboratory in the same week (Monday to Friday). Workshops were organised every trimester to improve the surveillance system, and site supervision targeted clinical personnel.

The National Influenza Reference Laboratory provided weekly reports on the distribution of the samples and the number of confirmed influenza cases to the Ministry of Health, to the sentinel sites and to WHO FluNet (http://www.who.int/influenza/gisrs_laboratory/flunet/en/).

### Laboratory procedures

Three aliquots were taken from each sample, two of which (1 ml each) were stored at −80 °C for external quality assessment (Centre for Health Protection, Department of Health, Hong Kong) and further analysis. The other (140 μl) was kept at 4 °C for RNA extraction with the QIAmp Viral RNA Mini Kit (QIAagen, Courtaboeuf, France) according to the manufacturer’s protocol. Influenza virus was detected and subtyped by real-time and multiplex reverse transcription polymerase chain reaction (RT-PCR) within 72 h of sample reception [20].

### Data management and analysis

Data from completed questionnaires and laboratory results were captured with EpiInfo, version 7. To estimate disease prevalence according to age, we categorised the patients by age: 0 to <6 months, 6 months to <1 year, 1 to <2 years, 2 to <5 years, 5 to <15 years, 15 to <50 years, 50 to <65 years and ≥65 years [18]. The demographic characteristics of all patients and of positive cases according to age and the seasonal patterns of influenza virus circulation were analysed with Stata version 12 (StataCorp, Texas, USA). The chi-squared test was used to assess differences in proportion and analysis of variance (ANOVA) to compare the average ages of patients between sentinel sites. A *P* value <0.05 was considered statistically significant.

## Results

### Characteristics of the study population

Of the 5385 patients enrolled during the study period, 2587 (48.0%) were male, and 2939 were enrolled from the sentinel sites in Bangui. The average age of the studied population was 9.0 years (SD, 13.5 years; range, 1 month to 82 years); children aged <5 years accounted for 61.4% and people ≥65 years of age for 0.3%. The distribution of the study population by demographic characteristics is shown in Table 1.

**Table 1:** Demographic characteristics of the population enrolled in the influenza sentinel surveillance system, Central African Republic, 2010–2015

	Bangui n (%)	Boali n (%)	Bossembele n (%)	Pissa n (%)	Sibut n (%)	Total N (%)
Number of patients	2939	616	856	870	104	5385
Sex						
Male	1492 (50.8)	289 (46.9)	379 (44.3)	385 (44.2)	42 (40.4)	2587 (48.0)
Female	1447 (49.2)	327 (53.1)	477 (55.7)	485 (55.8)	62 (59.6)	2798 (52.0)
Age						
Mean ± SD (years)	6.0 ± 10.1	17.3 ± 17.4	11.8 ± 15.5	10.5 ± 14.5	11.6 ± 16.6	9.0 ± 13.5
0 to <6 months	685 (23.3)	42 (6.8)	97 (11.3)	121 (13.9)	10 (9.6)	955 (17.7)
6 months to <1 year	396 (13.5)	59 (9.6)	100 (11.7)	99 (11.4)	13 (12.5)	667 (12.4)
1 to <2 years	198 (6.7)	30 (4.9)	47 (5.5)	62 (7.1)	11 (10.6)	348 (6.5)
2 to <5 years	761 (25.9)	97 (15.7)	232 (27.1)	216 (24.8)	28 (26.9)	1334 (24.8)
5 to <15 years	522 (17.8)	107 (17.4)	128 (14.9)	143 (16.4)	13 (12.5)	913 (16.9)
15 to <50 years	350 (11.9)	231 (37.5)	214 (25.0)	200 (23.0)	23 (22.1)	1018 (10.9)
50 to <65 years	20 (0.7)	48 (7.8)	35 (4.1)	26 (3.0)	6 (5.8)	135 (2.5)
≥ 65 years	7 (0.2)	2 (0.3)	3 (0.4)	3 (0.3)	0 (0.0)	15 (0.3)

### Influenza viruses and temporal distribution

Influenza virus A or B was found in 8.4% of samples that tested positive for influenza, and co-infection with A and B viruses was detected in four samples, for a total of 454 strains of influenza virus detected. The predominant influenza A viruses (55.7%; 253); 76 (16.7%) were A/H1N1pdm09, while 177 (39.0%) were influenza A/H3N2 and 201 (44.3%) were influenza B (Table 2).

**Table 2:** Distribution of influenza virus infection by demographic characteristics, Central African Republic, 2010–2015

Demographic characteristic	Influenza cases n (%)		P	Influenza virus identified n (%)					P^a^
	Negative	Positive		A/H1N1pdm09	A/H3N2	B	A/H1N1pdm09 + B	A/H3N2 + B	
Sex									
Male	2363 (91.3)	224 (8.7)	0.441	35 (15.6)	88 (39.3)	99 (44.2)	-	2 (0.9)	**0.9329**
Female	2572 (91.9)	226 (8.1)		40 (17.7)	86 (38.0)	98 (43.4)	1 (0.4)	1 (1.4)	
Age									
0 to <6 months	871 (91.2)	84 (8.8)	0.02	15 (17.8)	24 (28.6)	45 (53.6)	-	-	**0.0359**
6 months to <1 year	620 (92.9)	47 (7.1)		7 (14.9)	16 (34.0)	24 (51.1)	-	-	
1 to <2 years	329 (94.2)	20 (5.7)		0 (0.0)	8 (40.0)	11 (55.0)	-	1 (5.0)	
2 to <5 years	1228 (92.0)	106 (7.9)		22 (20.7)	34 (32.1)	50 (47.2)	-	-	
5 to <15 years	841 (92.1)	72 (7.9)		12 (16.6)	38 (52.8)	21(29.2)	1 (1.4)	-	
15 to <50 years	906 (89.0)	112 (11.0)		15 (13.4)	53 (47.3)	42 (37.5)	-	2 (1.8)	
50 to <65 years	126 (93.3)	9 (6.7)		4 (44.5)	1 (1.0)	4 (44.5)	-	-	
≥ 65 years	15 (100)	0 (0.0)		-	-	-	-	-	
Sentinel site									
Bangui	2670 (90.8)	269 (9.1)	< 0.001	45 (16.7)	100 (37.2)	123 (45.7)	1 (0.4)	-	**0.0268**
Boali	547 (88.5)	71 (11.5)		17 (23.9)	23 (32.4)	28 (39.4)	-	3 (4.2)	
Bossembele	815 (95.2)	41 (4.8)		7 (17.1)	10 (24.4)	24 (58.5)	-	-	
Pissa	812 (93.3)	58 (6.7)		5 (8.6)	32 (55.2)	21 (36.2)	-	-	
Sibut	93 (89.4)	11 (10.6)		1 (9.1)	9 (81.8)	1 (9.1)	-	-	
Total	**4935 (91.6)**	**450 (8.4)**		**75 (16.7)**	**174 (38.7)**	**197 (43.7)**	**1 (0.2)**	**3 (0.7)**	

^a^ comparison of proportion of influenza A and influenza B according to demographic characteristics

Most influenza cases (8.8%) were found in infants aged 0–6 months. The prevalence of infection with influenza A/H1N1 pdm09 was highest in children aged 0–5 years and adults ≥65 years, while the rate of infection with A/H3N2 was higher in children aged 1–2 years. Infection, and influenza B viruses occurred predominantly in children aged <5 years.

**Fig. 2:**
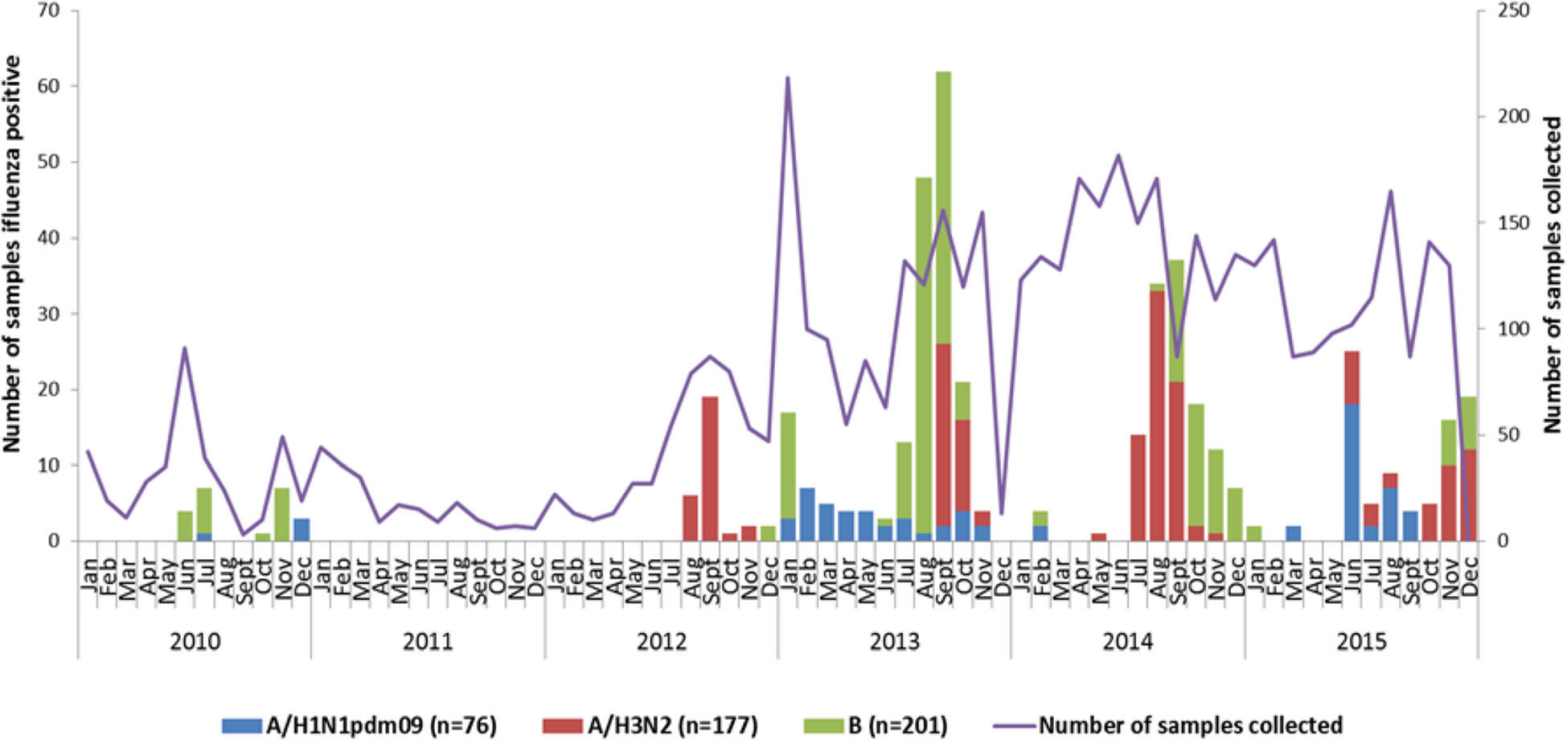
Monthly distribution of number of samples and influenza viruses detected, Central African Republic, 2010–2015

Irregular peaks of ILI were seen during the study period. Fewer samples were provided in 2010, 2011 and the first trimester of 2012 than in the two subsequent years. The number of cases of ILI increased markedly from July 2012, with peak activity varying annually. Influenza viruses were detected mainly during the rainy season (between July and December), influenza A/H3N2 and B being the most frequent. Overall, influenza B predominated in 2010 and 2013, while regular peaks of influenza A/H3N2 were observed between 2012 and 2015. H1N1pdm09 infection was first found in July 2010, circulated throughout 2013, was not detected in 2014 and reappeared in March–September 2015 with a peak in June (Fig. 2).

## Discussion

This first report of the results of sentinel surveillance for ILI in the Central African Republic shows that the influenza virus circulates, with peak activity during the rainy season (July–December). Although the seasonality of influenza viruses in African countries is not yet understood, we observed trends similar to those in other studies in tropical countries. This finding corroborates that of other countries that report increasing activity of influenza during the rainy season [11, 14, 21–23]. It has been argued that transmission of influenza viruses is influenced by increased contact due to promiscuity during the rainy season, when people mainly stay indoors [11]; however, in some tropical zones, seasonal patterns are less pronounced, with year-round detection of virus [13]. Conflicting results have been reported on the association between climate and influenza seasonality, and increased influenza transmission in the dry season may be due to enhanced viral transmission with low humidity [24]. It is surprising that the positivity rate for influenza virus found in our study was lower than those reported in Africa before 2010 [7, 11, 13, 14, 25] and during the same period as this study [12]. The main reason for the differences in percentage positive between our findings and those of other countries is the temporal distribution of influenza viruses [14].

We also found a significant discrepancy from other studies in the influenza virus-positive rates by age. Our study shows relatively high percentages of influenza cases in infants aged 0–6 months (8.8%) and in people aged between 15 and 50 years (11.0%), while other studies reported the highest rates of influenza viral infection in children aged 5–14 years and the lowest proportion among those aged 0–4 years [7, 14]. It has been proposed that the proportion of influenza viral disease in children <6 months of age is lower because of active transport of influenza-specific maternal antibodies across the placenta [26], although this distribution pattern is not universal [27].

The distribution of pandemic influenza A/H1N1pdm09 was highest in children <5 years, as seen in other studies [14, 16, 28]. We found that influenza B virus infected mainly children <5 years, although people aged 15–50 years generally had high proportions. Maman et al. in Togo also reported that influenza B infections occurred more frequently among older children and young adults [14].

The study has some limitations. First, the data were collected only at sentinel sites in Bangui and surrounding districts and cannot be generalised to the entire population of the country. Moreover, children were over-represented, particularly in Bangui, where the two main sentinel sites provide health care mainly to children. Another limitation was that surveillance was conducted only on outpatients, whereas it will be essential to report the proportion of severe acute respiratory infection associated with influenza viruses in hospitalised cases.

## Conclusions

Our findings contribute to understanding of the epidemiology of influenza in Africa. Influenza viruses circulate mainly during rainy season. We found that pandemic influenza A/H1N1pdm09 is circulating in the Central African Republic. Sentinel surveillance should be extended to other settings in the country, and the 91.0% of samples that test influenza-negative should be tested to identify other viral and bacterial etiologies. Furthermore, sentinel surveillance of severe acute respiratory illness should be conducted.
